# Application of the Just-About-Right Scales in the Development of New Healthy Whole-Wheat Muffins by the Addition of a Product Obtained from White and Red Grape Pomace

**DOI:** 10.3390/foods8090419

**Published:** 2019-09-17

**Authors:** Miriam Ortega-Heras, Inmaculada Gómez, Sara de Pablos-Alcalde, María Luisa González-Sanjosé

**Affiliations:** Facultad de Ciencias, Plaza Misael Bañuelos s/n, 09001 Burgos, Spain; miriorte@ubu.es (M.O.-H.); sda0016@alu.ubu.es (S.d.P.-A.); marglez@ubu.es (M.L.G.-S.)

**Keywords:** muffins, JAR, sensory properties

## Abstract

The aim of this study was to evaluate the adequacy of sensory attributes, by the use of just-about-right (JAR) scales and penalty analysis, of new healthy whole-wheat muffins with high fibre content due to the addition of two products obtained from white and red grape pomace. Furthermore, the nutritional, colour and texture properties of the muffins were evaluated. For that, five formulations of whole-wheat muffins were studied: a control muffin made with 100% whole-wheat flour and muffins made with 10 and 20% of white and red grape pomace product, respectively. The incorporation of grape pomace products in the recipe led to muffins with "high-fibre content", which would imply healthy properties in these muffins. The hardness and chewiness increased whereas the springiness, cohesiveness, resilience and colour parameters decreased when the grape pomace products were incorporated. The sensory analysis indicated high levels of acceptability of the muffins that incorporated white and red grape pomace products at concentrations of 10%. The penalty analysis showed that none of the attributes evaluated affected the acceptability of the new muffins significantly; only the darker colour of the muffins caused by the red grape pomace product could affect the acceptability, although this fact was not clearly demonstrated.

## 1. Introduction

Nowadays, there is a high demand for functional foods and the supply of healthier dietary options on the market is increasing. The reformulation of existing products is a viable alternative to develop new products. In fact, many healthier foods have been developed by the addition of functional ingredients, improving original recipes. However, the modifications induced by the reformulation do necessitate the evaluation of different aspects to assert the new product’s success. Firstly, the cost of production should be reasonable and have low repercussion on the final price of the new product. Furthermore, safety should not be altered, shelf life should not be reduced and sensory properties should be satisfactory to consumers. In summary, the new product has to meet consumers´ needs and expectations. For that reason, its acceptability has to be evaluated [1]. In addition, the knowledge about the consumers’ perception of the different sensory attributes and of the contribution of each attribute to the acceptability is a very useful information to identify potential improvements of the recipe to be moved closer to the “ideal” product [2].

In classic sensorial science, sensory characterization has been traditionally carried out by trained judges. However, the training and maintaining a sensory panel can be quite expensive and time consuming. For that reason, sensory sciences have developed more flexible and rapid sensory tools that give extra agility to sensory characterization, both in terms of timing and training requirements [3]. Some of these new techniques are based on the evaluation of individual attributes such as Check-All-That Apply (CATA) and flash profiling. Other methods are based on the evaluation of global differences, as with sorting and mapping; others still, such as just-about-right scales (JAR), not only describe the product but also allow to determine the ideal level of the product attributes [4]. When the JAR scales are used, consumers are asked to rate the intensity of an attribute, indicating whether the intensity of it is just about right, too strong or too weak compared to their internal ideal [5].

Within the most-used techniques to get information about consumers’ perception of the sensory attributes of a product are the JAR scales and attribute liking questions [2]. The JAR scales usually have five points to assess whether there is much less, much more or a “just-about-right” level of an attribute [6] and are a reliable tool to study the adequacy of sensory attributes [7]. Furthermore, it should be noted that a penalty analysis is used in order to gain an understanding of the attributes that most affected liking ratings [8]. This method provides guidance for product reformulation or a better understanding of attribute adequacy in relation to liking in terms of direction, with the assumption that the maximum hedonic score will occur at the “just-about-right” point [9,10].

Muffins are one of the most frequently consumed bakery products. Moreover, fibre is, among others, a healthy functional ingredient that has had a high impact over recent decades [11]; therefore, adding fibre to muffins without affecting the sensory characteristics is a significant challenge. Different studies have determined that grape pomace, a winemaking by-product, is an excellent source of dietary fibre to use in nutraceutical, medical and alimentary applications [12,13]. There are numerous food matrices that incorporate different additives obtained from white and red grape pomace (bread, biscuits, muffins, yoghurt, cheese, sausages, seafood, purée, etc.) at levels that range between 0.1% in cheese to 100% in infusions [14]. However, one of the problems associated with the incorporation of these products in food matrices is that they can modify sensorial properties [14]. In this sense, Acun and Gül [15] demonstrated that the use of flours prepared with grape-skin extract at concentrations of 5% in biscuits improved their acceptability, but high levels of this extract led to consumer rejection, due to a bitter taste and a darkening of the product. Therefore, when products obtained from winemaking by-products are used in the reformulation of new products, it is necessary to determine the maximum acceptable dose and to evaluate all those organoleptic properties that can affect the global acceptability of the product by the consumer.

It should be highlighted that, although recent consumer trends have embraced the concepts of sustainability and health, the sensory properties of foods are the most important reason people eat the foods they eat [16]. Therefore, the main aim of this work was to evaluate the sensory properties and adequacy of sensory attributes, by the use of JAR scales and penalty analysis, of new healthy whole-wheat muffins. Furthermore, their nutritional, colour and texture properties were evaluated.

## 2. Materials and Methods 

### 2.1. Ingredients

The ingredients used in the preparation were whole-wheat flour, sunflower oil, brown sugar, semi-skimmed milk, baking powder and salt, which were obtained from a local supermarket.

The products used in this study were obtained from white and red grape pomace and were named WP (white product) and RP (red product), respectively. They were obtained according to the patented method CCP:ES2524870 [17]. Briefly, dried grape pomace free of seed was milled, sieved (particle size <250 µm mesh) and microbiologically stabilized, obtaining powder products useful to be used as food ingredients. Obtained products were kept under vacuum and darkness until their use. The composition of the RP was: total dietary fibre (48.6%), total protein (14.4%), total lipid (3.7%) and ash (14.4% of dry matter; potassium: 43.3 mg/g of dry matter; total phenolic content: 25.9 mg gallic acid/g) [9]. Moreover, the composition of the WP was: total dietary fibre (53.3%), total protein (14.4%), total lipid (3.7%) and ash (9.8% of dry matter; potassium: 36.5 mg/g of dry matter; total phenolic content: 13.8.9 mg gallic acid/g) [18].

### 2.2. Elaboration of the Muffins

The following muffins were prepared: a control muffin with 100% of whole-wheat flour (C) and muffins with 10 and 20% of each grape pomace product, white (WP-10 and WP-20) and red (RP-10 and RP-20), replacing the part corresponding to whole-wheat flour with the product. 

The formulation employed in each muffin is listed in Table 1.

The different formulations of muffins were prepared with a mixer (KitchenAid KSM90, Benton Harbor, MI, United States). Firstly, the eggs were beaten until they peaked. Then, the milk and the sunflower oil were quickly added, ensuring that all the ingredients were well mixed. Subsequently, the mixture of whole-wheat flour, yeast, and the seasoning, or the salt in the case of the control muffins, were added and beaten at high speed for 5 min. Finally, the mixture was beaten at high speed for 3 min and spooned into paper baking cups. The selected baking temperature was 180 °C for 17 min. After baking, the samples underwent packaging with a partial vacuum of 30% in polypropylene bags and they were stored at room temperature for 24 h until the corresponding analysis.

Three batches, each one with 40 muffins, of each of the different formulations of the study were prepared on three different days.

### 2.3. Nutritional Composition 

Moisture content was determined by drying at 130 °C for 90 min. The fat content was evaluated by Soxhlet extraction using petroleum ether in a Buchi B-811 extraction system (Büchi, Flawil, Switzerland). The dry and defatted residues were used for the analysis of protein and total dietary fibre contents. The protein content was determined using the Kjeldahl method with a conversion factor of 5.70 to convert nitrogen into protein values. The total dietary fibre was determined by the AOAC enzymatic-gravimetric method [19,20]. The enzymatic kit was supplied by Sigma (St Louis, MO, USA). In each batch, all these analyses were carried out by duplicate.

### 2.4. Height Increase and Weight Loss 

Muffins’ height and weight are some of the most common parameters used to control the baking process and the homogeneity among lots. Furthermore, they are indirect indicators of some muffins’ quality parameters as sponginess and firmness.

Muffins were weighed and their height was measured before going in the oven and after baking and cooling for 1 h according to the method proposed by Martínez-Cervera et al. [21]. Weight loss and height increase were calculated by difference between both weights and heights. Three different samples from each batch and formulation were measured.

### 2.5. Colour

Instrumental measurement of the colour of the muffins was performed at 24 h after baking, employing a Konica Minolta CM-2600d spectrophotometer (Konica Minolta Business Technologies Inc., Tokio, Japan). The D65 illuminance and 10º standard observe were selected. The colour coordinates were determined in the CIELAB colour space, expressing the results in terms of L* (lightness), a* (redness) and b* (yellowness).

Crust and crumb colour were measured by duplicate on five different muffins by batch and formulation. For the measure of the crumb colour, each muffin was cut in half parallel to the base.

### 2.6. Texture Profile Analysis (TPA)

The TPA of samples (2 × 2 × 2 cm) obtained from the mid-section of the muffins was measured using a TA.XT.plus Texture Analyzer (Stable Microsystem Ltd., Surrey, UK). A double compression cycle test was carried out with an aluminium 50 mm diameter cylinder probe and to 50% compression, at 1 mm/s speed test with 5 s of delay between the two cycles. The texture parameters (hardness, springiness, cohesiveness, chewiness and resilience) were calculated from the texture profile graphics. The texture parameters of each muffin formulation were averaged from nine measurements (three muffins for each batch). 

### 2.7. Sensory Analysis 

The standardized tasting room equipped with individual booths [22] of the Faculty of Science (Burgos University, Burgos, Spain) was the place where consumers tasted the muffins. A total number of 104 untrained judges, habitual consumers (who consumes muffins at least once every week) participated in the assays. They were students, professors and administrative employers of the University of Burgos, with ages ranged between 18 and 55 years (69% female, 31% male).

Sensory evaluations were carried out on three different days, which means that three different batches of each formulation were evaluated. Muffins were always evaluated 24 h after their elaboration. After consumers had read the informed consent form for sensory evaluation, they evaluated five muffins in a single session (one of each formulation). Samples were presented one-by-one, following a balanced complete block experimental design. The samples were coded with random three-digit numbers. Consumers could rinse their mouth with water between samples.

Consumers indicated the degree of liking of each muffin using a nine-point hedonic scale, ranged from 1 = “dislike extremely” to 9 = “like extremely” with middle value 5 = “neither like nor dislike”, whereas the adequacy of the six attributes (“surface colour”, “crumb colour”, “sweetness”, “hardness”, “sponginess” and “flavour”) was measured using “just-about-right” (JAR) bipolar scales of five points ranged (from 1 = “much less” to 5 = “much more” with middle value 3 = “just about right”). These scales are used in studies with consumers to establish whether a particular attribute is perceived in a product at an excessive, scarce or acceptable level. The extreme ends of the scale represent the level of an attribute that moves away from the ideal theoretical point in opposite directions, while the central point is the ideal or acceptable one [9]. Penalty analysis (PA) was carried out transforming the initial scale of five points into one of three points. For that, the responses “much less” and “less” were grouped into an unique group named “much less” and the responses “more” and “much more” were grouped in an unique group named “much more” [1,9,23,24].

### 2.8. Statistical Analysis

The results of physico-chemical and nutritional properties and liking scores were presented as the average ± standard deviation of the different replicates. An ANOVA test was performed with the purpose of determining whether statistically significant differences existed among the samples at a significance level of *p* = 0.05. The LSD (Least Significant Difference) test was performed with the objective of establishing between which samples there were statistically significant differences. The Statgraphics Centurion XVII.I software (IBM Corp., Armonk, NY, USA) program was used.

The JAR results were analysed by PA to identify potential directions for product improvement on the basis of consumer acceptability by highlighting the most penalizing attributes in liking terms [10].

## 3. Results

### 3.1. Nutritional Composition

Results showed that the incorporation of the grape pomace products caused significant changes in the nutritional composition of the muffins, although not all the studied parameters showed statistically significant differences (*p* < 0.05) (Table 2).

Moisture was similar in the five types of muffins, which can be explained because the grape pomace products used are not very hygroscopic. This result is contrary to those described by Bender et al. [25], who found higher moisture values in muffins elaborated with white grape-skins. However, the difference is easy to explain considering that they used products containing sugars, whereas the products used in this study were free of sugars. Values of protein content were not statistically different. However, it is possible to point out that qualitatively, muffins with red or white product showed a lower quantity of proteins. This fact agrees with a dilution effect, mainly due to the fibre contribution of the product (ranged from 48% to 53%, [12,18]). In fact, all the muffins elaborated with the products presented higher concentrations of fibre than the control muffin. These results agree with others found by others authors in different bakery products [15,25,26]. It should be highlighted that, according to Regulation (EC) No 1924/2006 of 20 December 2006 [27], the claim “high in fibre” may only be used if the product contains a minimum 6 g of fibre per 100 g or 3 g of fibre per 100 kcal. Therefore, the muffins elaborated with the grape pomace products could be labelled with the claim “high in fibre”.

The product addition also modified the fat levels, being higher in muffins elaborated with red or white pomace products. This fact is mainly due to the fat contribution of the grape product. Fat content of the product was ranged from 3.3 to 3.8% [12,18], which is higher that the fat content of the flour (1.8% according to brand ingredient information). Acun and Gül [15] observed similar results in biscuits fortified with a grape-skin extract.

### 3.2. Height and Weight Loss 

Results obtained showed that the incorporation of the products led to statistically significant difference (*p* < 0.5) in the height increase of the muffins (Table 3). 

Control muffins showed the highest height increase, which decreased as the percentage of added grape pomace product increased. This may be due to the fat and the fibre present in the seasoning, which affected the structure of the dough, having a negative influence on CO_2_ retention [28]. Similar results were obtained when date fruit were incorporated to muffins [29]. Regarding the percentage of weight loss (Table 3), a similar effect was observed in the muffins with a 20% of the product, leading to comparable results to those found by Bender et al. [25] when adding products obtained from red and white grape-skins in the elaboration of muffins.

### 3.3. Colour

Colour parameter values were statistically different (*p* < 0.5) among samples (Table 4). The muffins elaborated with the grape pomace products were darker than the control muffins, showing lower lightness values, both in the crust and in the crumb. A significant effect of the amount of the red grape pomace product was found; thus, the higher percentage of added RP, the lower lightness in the crust and crumb of muffins. The results obtained in terms of lightness were similar to those found by Walker et al. [28] and Bender et al. [25] when fortifying muffins with products obtained from red and white grape-skins. 

As with lightness, an effect of the type and the concentration of the product employed in the parameters a* and b* was found. Thus, the lowest values of these parameters corresponded to the muffins with red grape pomace product. In relation to the a* parameter in crumbs, the muffins with the product obtained from white grape pomace showed higher values of this parameter than the control ones. This may be due to the own colour of the white grape pomace product.

As can be seen, the combination of the a* and b* parameters gave as a result the colour of each sample. The a* and b* values obtained in the samples with white product were higher than those with the red product, giving the characteristic yellowness of whole-wheat muffins. However, lower values were related to the dark colour of the muffins with red product. The same tendency towards lower values of the a* and b* parameters was observed by Acun and Gül [15] in the preparation of biscuits with red grape-skin pomace extracts.

### 3.4. Texture

The addition of the two concentrations of products also modified the texture parameters evaluated (Table 5). The incorporation of the seasonings increased significantly (*p* < 0.05) the hardness of the muffins, which may be due to the higher fibre content of these samples, which increased the dough density and reduced the incorporation of air during baking. Thus, the resulting crumbs were more compact, which increased the force required to compress them [25]. In the case of the muffins with WP, it was also observed a relationship between the amount of added product and the hardness.

WP-20 and RP-20 muffins also underwent a significant reduction (*p* < 0.05) of springiness due to the higher levels of fibre, which increased the density of the samples, reducing their springiness [28]. Fibre also influenced on cohesiveness; thus, the values decreased as the percentage of grape pomace product added increased. The desegregation effect of the fibre is reflected in lower requirement of energy when TPA performs a second compression [30].

In relation to chewiness, no statistically significant differences were found among the muffins with grape pomace products, although their values were higher than those of the control muffin. 

As for the resilience, an effect of the percentage of seasoning added was found, decreasing the resilience value as the added amount of seasoning increased. All the samples studied showed low resilience values (0.218 ± 0.022), which are typical values of recipes with fat and sugar [25].

The results of the textural instrumental technique in the present study (Table 5) were similar to those found in the study by Walker et al. [28], in which the impact of the incorporation of red and white grape-skin in different products was evaluated with the aim of obtaining functional bakery dough. The same tendency was observed in the study performed by Bender et al. [25] on muffins.

### 3.5. Sensory Analysis

#### 3.5.1. Liking Study

**Figure 1:**
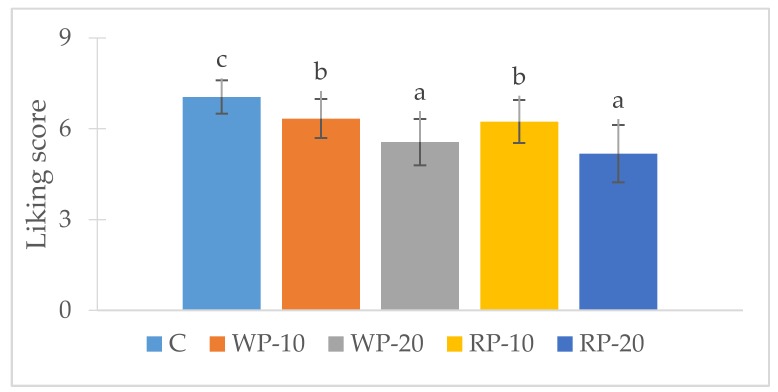
Mean consumer liking scores of the control (C) and reformulated muffins (*n* = 104). WP-10: muffin with 10% of white product (WP); WP-20: muffin with 20% of WP; RP-10: muffin with 10% of red product (RP); RP-20: muffin with 20% of RP. Different letters in the bars denote significant differences between products. LSD test and *p*-value < 0.05.

The consumer scores for overall-liking showed statistically significant (*p* < 0.05) differences among muffins (Figure 1). The highest score was for the control muffin (7.05 ± 1.11) followed by those of WP-10 and RP-10 muffins (6.34 ± 1.29 and 6.24 ± 1.42, respectively), being the lowest scores those of WP-20 and RP-20 muffins (5.56 ± 1.53 and 5.18 ± 1.89, respectively). Therefore, the incorporation of the grape product reduced the overall-liking significantly (*p* < 0.05). The lower overall-liking scores of the muffins prepared with 20% product could be related to changes in the taste and colour. Acu and Hul 2013 [15] also observed that the addition of more than a 5% of whole red grape pomace, seedless red grape pomace and seed to a cookie recipe led to a darkening of the crust, an increase of the hardness, and a loss of flavour and taste and, consequently, their acceptability decreased.

The results obtained in the sensory evaluation of the present study are similar to those obtained by Bender et al. [25]. However, Pitre et al. [31] reported an acceptability level of 67% without prompting any observation by the consumers for a biscuit prepared with 10% of grape-skin extract, and Mildner-Szkudlarz et al. [32] observed no significant differences in the acceptability of muffins added with less than 20% of white grape-skin extract.

As previously reported, the muffins elaborated with the grape pomace products could be labelled with the claim “high in fibre”. However, consumers’ main scepticism regarding functional foods resides in the veracity of health claims [33]. Thus, in order to obtain product acceptability, clear, understandable and verified information has to be communicated to the consumer [1]. Therefore, it would be needed to further investigate the willingness to purchase of consumers when the claim and information about healthy properties of the new muffins (with WP or RP) are provided to consumers, and thus, to know if the reduction of 0.76 points in the overall-liking scores of the WP-10 and RP-10 muffins could be assumed.

#### 3.5.2. Attribute Adequacy and Its Relation to Liking-Penalty Analysis 

**Figure 2:**
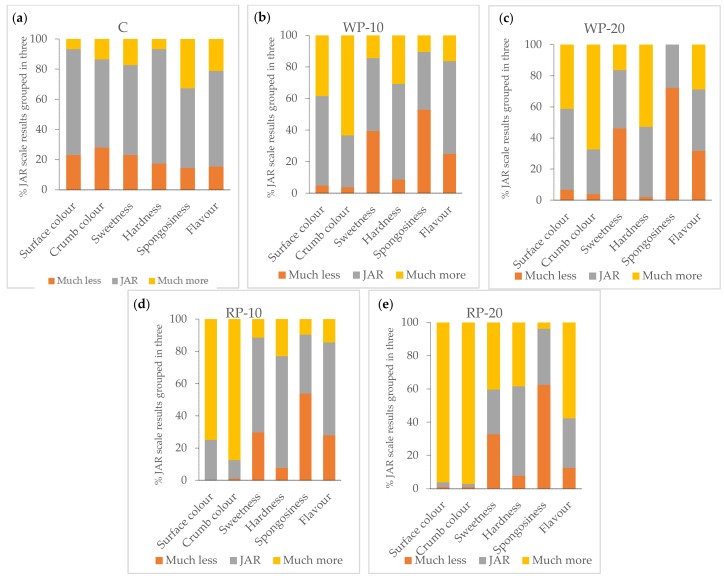
Just-about-right (JAR) scale percentages of responses grouped in three levels of the muffins (*n* = 104). (**a**) C: Control; (**b**) WP-10: muffin with 10% of white product (WP); (**c**) WP-20: muffin with 20% of WP; (**d**) RP-10: muffin with 10% of red product (RP); (**e**) RP-20: muffin with 20% of RP.

The results obtained for the JAR scales are shown in Figure 2. 

In the control muffins all the parameters were mainly scored in “JAR” category, ranging from 53% (sponginess) to 76% (hardness). The most remarkable results were that 33% of the consumers thought that the sponginess of the control muffins was “much more” and 28% indicated that the crumb colour was “much less”. The reformulated muffins showed lower percentages of JAR responses than the control muffins for most of the attributes evaluated (see Appendix A), and it was observed that the percentage of JAR responses dropped as the percentage of addition of the grape product increased.

The hardness was the parameter that showed lower differences with control muffins, especially when only 10% of grape pomace product was used. However, larger differences in scores were observed related to colour attributes, especially in muffins elaborated with the product from red grapes. In these cases, responses in the group “JAR” were lower than 25% in the RP-10 muffins, whereas in the RP-20 muffins, these percentages dropped to the 2%. Consumers considered these muffins to have “much more” colour. Furthermore, they also indicated that they had “much more” flavour (58%). The obtained results also showed that the white grape pomace product decreased the perception of sweetness with respect to the control muffin more than the red grape pomace product. Thus, in the WP-20 muffins, the percentage of responses “much less” was 23 points higher than that of the control muffins, whereas in the RP-10 muffins, this increase was only of 10 points.

An analysis of penalizations was carried out to understand which of the attributes under evaluation affected the acceptability of the product to a greater or a lesser extent. The penalizations indicate how much the global acceptability of a product drops when a particular attribute is seen as “much more” or “much less”, in such a way that the higher the values that are obtained, the greater the impact of the aforementioned acceptability [24]. The results are shown in Table 6. 

In those categories where the number of responses was below the threshold of 20%, the penalizations were not taken into account [34]. Moreover, an attribute was only considered to affect the acceptability of the product when the mean drop was higher than 1 [24].

Neither of the parameters had mean drops over 1 (data not shown), which means that there was no attribute that by itself affected the acceptability in a significant way. Then, results seemed to point out that the acceptability depended on the joint perception of all of them together. However, penalties could not be calculated for the colour of the crust and the crumb of the muffins elaborated with red grape pomace product; a high percentage of responses were obtained at the extreme point of “much more” on the scale: over 90% for a concentration of 20% seasoning. This fact highlighted that colour was one of the parameters furthest from the “ideal colour”. However, there was not a clear correlation of this fact with the overall-liking of the muffins, especially with those elaborated with red grape pomace product. Previous studies noted that muffins’ colour is a decisive factor in the global acceptability as well as in the purchase intention [35], however, the obtained results are not as robust. Thus, 88% of the consumers indicated that the acceptability of the crumb colour of the RP-10 muffins was "much more", whereas for the control muffins this percentage was 13%. However, the acceptability of RP-10 muffins was only 0.76 point lower than that of control muffins. This fact points out one of the problems of the JAR scales, which is that this methodology evaluates the degree of acceptability of each attribute in an individual way, without taking into account that the modification of an attribute can influence the perceptions of others in either a positive or in a negative manner [24].

Independently of previous comments, if the dark colour of the muffins elaborated with grape pomace product was actually an important problem for the consumer acceptability of the product, a possible solution could be to inform the consumers of the origin of the “dark colour”, explaining also the associated healthy effects of their components such as fibre, antioxidants (grapes phenolic compounds), minerals etc. In fact, consumers usually associated darker baked products with healthy properties and higher fibre and whole grain content [28]. In this way, if the new muffins were marketed specifying the healthy characteristics of the grape pomace product, the dark colour of the muffins elaborated with them should not be a reason for their initial rejection by consumers. Other studies have shown that providing information on the fibre content of muffins has a positive impact on consumer perception and acceptability, regardless of the appearance of the product [11].

## 4. Conclusions

The incorporation of grape pomace led to changes in the colour and textural properties of the new whole-wheat muffins with high content of fibre. However, the sensory analysis indicated a good level of acceptability for these new healthy whole-wheat muffins with the grape pomace products, although consumers indicated that some sensorial parameters were different from those considered as “ideal”. Moreover, the muffins with 10% of WP or RP had higher liking scores than those with 20% of WP or RP. However, in spite of the fact that a penalty analysis indicated that the darker surface colour in muffins with RP could affect the acceptability, the advice of the use of RP in the formulation and its healthy properties could overcome this problem. Thus, further studies would be needed in which information on health properties is presented to the consumers during the evaluation to fully understand the willingness to purchase of new healthier products. Furthermore, the information obtained from the JAR scales and penalty analysis indicated that grape pomace products obtained from red and white grapes at a level of 10% could be a good alternative to develop new healthier muffins with high fibre content.

## Figures and Tables

**Table 1:** List of the ingredients of the control (C) and the reformulated muffins.

Ingredients (g)	C	WP-10	WP-20	RP-10	RP-20
Whole-wheat flour	210	189	168	189	168
Brown sugar	175	175	175	175	175
Sunflower oil	190	190	190	190	190
Semi-skimmed milk	60	60	60	60	60
Eggs (units)	4	4	4	4	4
Chemical yeast	8	8	8	8	8
WP	-	21	42	-	-
RP	-	-	-	21	42
Salt	5.2	-	-	-	-

WP-10: muffin with 10% of white product (WP); WP-20: muffin with 20% of WP; RP-10: muffin with 10% of red product (RP); RP-20: muffin with 20% of RP.

**Table 2:** Nutritional composition (mean ± standard deviation) of the control (C) and the reformulated muffins.

Sample	Moisture (%)	Protein (%)	Fat (%)	Total Dietary Fibre (g/100 g)
C	14.4 ± 2.3 ^a^	6.92 ± 1.81 ^a^	26.7 ± 2.2 ^a^	5.67 ± 0.44 ^a^
WP-10	14.2 ± 0.9 ^a^	5.77 ± 0.46 ^a^	31.0 ± 3.9 ^b^	9.56 ± 0.55 ^c^
WP-20	14.1 ± 2.3 ^a^	5.99 ± 0.96 ^a^	32.4 ± 1.2 ^bc^	11.9 ± 0.3 ^d^
RP-10	14.7 ± 2.8 ^a^	5.54 ± 0.33 ^a^	33.3 ± 1.9 ^bc^	8.24 ± 0.64 ^b^
RP-20	14.7 ± 3.9 ^a^	5.42 ± 1.19 ^a^	33.8 ± 1.1 ^c^	11.2 ± 0.7 ^d^

WP-10: muffin with 10% of white product (WP); WP-20: muffin with 20% of WP; RP-10: muffin with 10% of red product (RP); RP-20: muffin with 20% of RP. Different letters in the same column denote significant differences between products. Least Significant Difference (LSD) test and *p*-value < 0.05.

**Table 3:** Height increase (mm) and weight loss (%) (mean ± standard deviation) of the control (C) and the reformulated muffins.

Sample	Height Increase (mm)	Weight Loss (%)
C	23.0 ± 2.6 ^c^	15.1 ± 0.6 ^b^
WP-10	13.9 ± 2.3 ^b^	15.4 ± 0.9 ^b^
WP-20	8.00 ± 5.81 ^a^	14.8 ± 1.7 ^ab^
RP-10	14.1 ± 3.9 ^b^	15.6 ± 0.9 ^b^
RP-20	11.4 ± 5.6 ^ab^	13.8 ± 1.4 ^a^

WP-10: muffin with 10% of white product (WP); WP-20: muffin with 20% of WP; RP-10: muffin with 10% of red product (RP); RP-20: muffin with 20% of RP. Different letters in the same column denote significant differences between products. LSD test and *p*-value < 0.05.

**Table 4:** Colour parameters (mean ± standard deviation) of the control (C) and the reformulated muffins.

	Sample	L*	a*	b*
Crust	C	35.6 ± 1.3 ^d^	12.4 ± 0.2 ^e^	20.1 ± 2.2 ^e^
	WP-10	33.2 ± 2.2 ^c^	10.7 ± 0.4 ^d^	15.5 ± 1.4 ^d^
	WP-20	31.8 ± 3.0 ^c^	9.76 ± 0.37 ^c^	14.2 ± 1.3 ^c^
	RP-10	29.0 ± 4.0 ^b^	7.10 ± 0.62 ^b^	10.8 ± 0.5 ^b^
	RP-20	26.3 ± 4.1 ^a^	4.30 ± 0.15 ^a^	6.14 ± 0.02 ^a^
Crumb	C	45.4 ± 3.2 ^d^	6.19 ± 0.29 ^c^	18.9 ± 0.7 ^d^
	WP-10	37.3 ± 0.8 ^c^	6.87 ± 0.36 ^d^	14.9 ± 1.0 ^c^
	WP-20	35.6 ± 3.9 ^bc^	7.24 ± 0.74 ^d^	14.6 ± 1.6 ^c^
	RP-10	33.9 ± 2.6 ^b^	4.22 ± 0.21 ^b^	8.85 ± 0.68 ^b^
	RP-20	29.7 ± 4.8 ^a^	3.83 ± 0.47 ^a^	5.79 ± 1.25 ^a^

WP-10: muffin with 10% of white product (WP); WP-20: muffin with 20% of WP; RP-10: muffin with 10% of red product (RP); RP-20: muffin with 20% of RP. Different letters in the same column denote significant differences between products. LSD test and *p*-value < 0.05.

**Table 5:** Texture parameters (mean ± standard deviation) of the control (C) and the reformulated muffins.

Sample	Hardness (*n*)	Springiness	Cohesiveness	Chewiness (*n*)	Resilience
C	5.56 ± 1.82 ^a^	0.843 ± 0.020 ^b^	0.659 ± 0.009 ^c^	3.08 ± 1.02 ^a^	0.248 ± 0.016 ^c^
WP-10	12.2 ± 2.2 ^ab^	0.838 ± 0.019 ^b^	0.609 ± 0.009 ^b^	6.19 ± 1.09 ^b^	0.224 ± 0.006 ^b^
WP-20	21.6 ± 14.9 ^c^	0.795 ± 0.024 ^a^	0.541 ± 0.014 ^a^	9.10 ± 5.88 ^b^	0.194 ± 0.014 ^a^
RP-10	15.1 ± 9.0 ^bc^	0.834 ± 0.011 ^b^	0.604 ± 0.015 ^b^	7.55 ± 4.35 ^b^	0.225 ± 0.018 ^b^
RP-20	17.3 ± 8.6 ^bc^	0.796 ± 0.038 ^a^	0.561 ± 0.081 ^a^	7.42 ± 2.76 ^b^	0.197 ± 0.033 ^a^

WP-10: muffin with 10% of white product (WP); WP-20: muffin with 20% of WP; RP-10: muffin with 10% of red product (RP); RP-20: muffin with 20% of RP. Different letters in the same column denote significant differences between products. LSD test and *p*-value < 0.05.

**Table 6:** Penalty analysis of the control (C) and the reformulated muffins.

Sample	Attribute	Level	% Consumers	Overall Liking
C	Surface colour	Much less	23	7.03
		JAR	70	7.05
		Much more	7	7.28
	Crumb colour	Much less	28	7.03
		JAR	59	7.05
		Much more	13	7.03
	Sweetness	Much less	23	7.02
		JAR	60	7.05
		Much more	17	7.30
	Hardness	Much less	17	7.05
		JAR	76	7.03
		Much more	7	7.05
	Sponginess	Much less	14	7.11
		JAR	53	7.03
		Much more	33	7.05
	Flavour	Much less	15	7.09
		JAR	64	7.05
		Much more	21	7.03
WP-10	Surface colour	Much less	5	6.26
		JAR	57	6.34
		Much more	38	6.34
	Crumb colour	Much less	4	6.30
		JAR	33	6.35
		Much more	63	6.29
	Sweetness	Much less	39	6.35
		JAR	46	6.34
		Much more	15	6.30
	Hardness	Much less	9	6.30
		JAR	60	6.36
		Much more	31	6.23
	Sponginess	Much less	53	6.30
		JAR	36	6.29
		Much more	11	6.40
	Flavour	Much less	25	6.25
		JAR	59	6.34
		Much more	16	6.34
WP-20	Surface colour	Much less	7	5.60
		JAR	52	5.56
		Much more	41	5.58
	Crumb colour	Much less	4	5.51
		JAR	29	5.56
		Much more	67	5.58
	Sweetness	Much less	46	5.57
		JAR	38	5.59
		Much more	16	5.60
	Hardness	Much less	2	3.33
		JAR	45	5.56
		Much more	53	5.58
	Sponginess	Much less	72	5.58
		JAR	28	5.59
		Much more	0.00	
	Flavour	Much less	32	5.56
		JAR	39	5.60
		Much more	29	5.59
RP-10	Surface colour	Much less	0	
		JAR	25	6.33
		Much more	75	6.24
	Crumb colour	Much less	1	4.00
		JAR	12	6.33
		Much more	87	6.24
	Sweetness	Much less	30	6.25
		JAR	59	6.29
		Much more	11	6.39
	Hardness	Much less	8	6.53
		JAR	69	6.29
		Much more	23	6.24
	Sponginess	Much less	54	6.24
		JAR	36	6.31
		Much more	10	6.38
	Flavour	Much less	28	6.25
		JAR	58	6.30
		Much more	14	6.38
RP-20	Surface colour	Much less	0.96	8.00
		JAR	2.88	5.05
		Much more	96.2	5.18
	Crumb colour	Much less	1	6.00
		JAR	2	3.57
		Much more	9	5.18
	Sweetness	Much less	33	5.12
		JAR	27	5.18
		Much more	40	5.22
	Hardness	Much less	8	5.10
		JAR	54	5.13
		Much more	39	5.19
	Sponginess	Much less	63	5.18
		JAR	34	5.16
		Much more	4	5.12
	Flavour	Much less	12	5.12
		JAR	30	5.19
		Much more	58	5.21

WP-10: muffin with 10% of white product (WP); WP-20: muffin with 20% of WP; RP-10: muffin with 10% of red product (RP); RP-20: muffin with 20% of RP.

**Figure A1:**
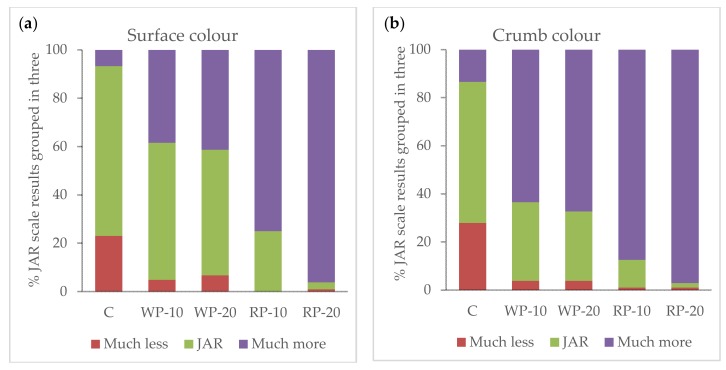

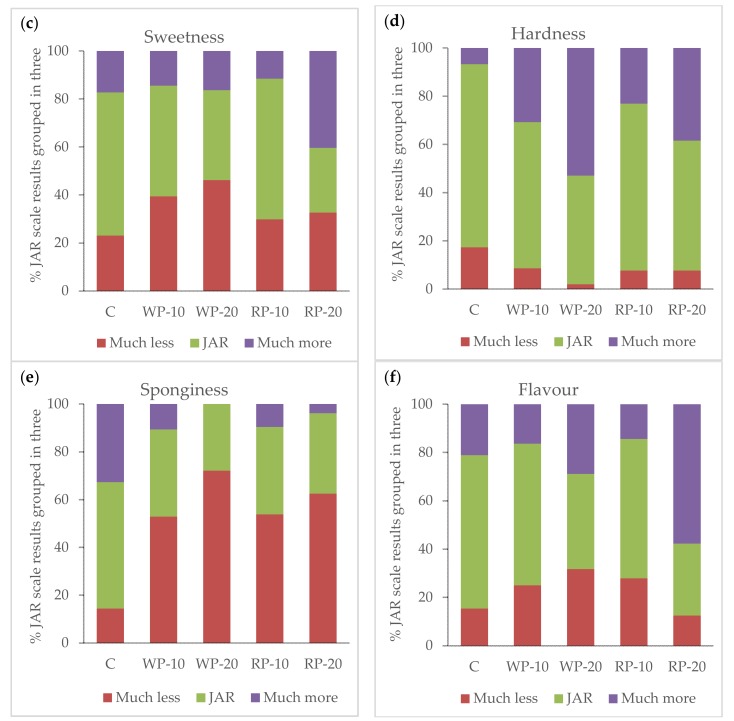
JAR scale percentages of responses grouped in three levels of the control (C) and the reformulated muffins for each attribute (*n* = 104). WP-10: muffin with 10% of white product (WP); WP-20: muffin with 20% of WP; RP-10: muffin with 10% of red product (RP); RP-20: muffin with 20% of RP. Parameters evaluated: (**a**) surface colour; (**b**): crumb colour; (**c**): sweetness; (**d**): hardness; (**e**): sponginess; (**f**): flavour.
